# Vaccination of children against COVID-19: the experience in Latin America

**DOI:** 10.1186/s12941-022-00505-7

**Published:** 2022-03-25

**Authors:** Alfonso J. Rodriguez-Morales, Darwin A. León-Figueroa, Luccio Romaní, Timothy D. McHugh, Hakan Leblebicioglu

**Affiliations:** 1 Grupo de Investigación Biomedicina, Faculty of Medicine, Fundación Universitaria Autónoma de las Américas, Pereira, Risaralda Colombia; 2 Master of Clinical Epidemiology and Biostatistics, Universidad Científica del Sur, Lima, Peru; 3 School of Medicine, Universidad Privada Franz Tamayo (UNIFRANZ), Cochabamba, Bolivia; 4 Facultad de Medicina Humana, Universidad de San Martín de Porres, Chiclayo, Peru; 5 Sociedad Científica de Estudiantes de Medicina Veritas (SCIEMVE), Chiclayo, Peru; 6 Centro de Investigación en Atención Primaria en Salud, Universidad Peruana Cayetano Heredia, Lima, Peru; 7 Emerge, Unidad de Investigación en Enfermedades Emergentes y Cambio Climático, Facultad de Salud Pública y Administración, Universidad Peruana Cayetano Heredia, Lima, Peru; 8 UCL Centre for Clinical Microbiology, Royal Free Campus, UCL, London, UK; 9 Department of Infectious Diseases, VM Medicalpark Samsun Hospital, Samsun, Turkey

The Coronavirus Disease 2019 (COVID-19) caused by the Severe Acute Respiratory Syndrome Coronavirus 2 (SARS-CoV-2) has spread globally, becoming a long-lasting pandemic [1–3]. As is so often the case for infectious diseases, vulnerable communities are likely to demonstrate the worse effects and this holds true for COVID-19 in Latin America [4].

Early in the pandemic, it was believed that COVID-19 did not significantly affect children. However, since the first confirmed pediatric case of COVID-19 was reported in Shenzhen, China, many cases have been reported and studied in pediatric patients [5]. It is now known that COVID-19 can affect children of all ages [6–9]. Although in many settings children usually have a lower risk of exposure and are tested less frequently than adults, the incidence in some countries in children is similar to that in adults [10].

During the surveillance in several countries, children typically account for up to 16% of laboratory-confirmed cases or even more, depending on the vaccinated groups. For example, in the United States, total childhood cases of COVID-19 reported since the beginning of the pandemic were 12.7 million cases, accounting for 19.0% of all cases [11].

In early reports of the disease in children, most cases resulted from exposure to SARS-CoV-2 within the home from contact with an adult carrier [7, 12, 13]. However, social gatherings with people outside the home and meeting other children at play activities were also associated with the transmission of the virus [14]. In addition, transmission related to health care and school attendance has also been reported [15–18].

COVID-19 infection mainly targets older people with comorbidities; children infected with SARS-CoV-2 have similar symptoms as adults; however, they have a milder course of illness and a better prognosis than adults, requiring less hospital admission [19, 20]. The primary infection characteristics in pediatric patients are fever and cough; diarrhoea, vomiting, nasal congestion, and fatigue may be found in a lesser proportion [21]. Forty-two per cent of the cases could generate an asymptomatic clinical picture, and 3% require hospitalisation [22]. Multisystem Inflammatory Syndrome in Children (MIS-C) is a severe complication of exposure to SARS-CoV-2 viruses, which may require admission to intensive care, mechanical ventilation, and cardiorespiratory support. However, it rarely leads to death [23, 24]. This clinical syndrome is characterised by fever, systemic inflammation, and multisystem involvement, most commonly abdominal and cardiac, apparently driven by an uncontrolled immune response [24, 25].

Immunisation is the most effective public health strategy against the SARS-CoV-2 pandemic [26]. Vaccines protect children and reduce the spread of disease to families and communities; given the lower risk of severe COVID-19 in young children, vaccine safety is paramount, monitored by the Centers for Disease Control and Prevention and other national or regional agencies [27].

Early in the pandemic, there was a compelling need to quickly develop vaccines in less than a year to prevent the viral spread and save lives [28]. The COVID-19 vaccine is necessary to achieve herd immunity and is essential to mitigate the spread of the pandemic [29].

Currently, there are 9 vaccines with greater than 50% efficacy against symptomatic COVID-19 in adults: with 96% we have NVX-CoV2373 (Novavax, USA), with 95% BNT162b2 (Pfizer/BioNTech, USA & Germany), with 94.1% mRNA-1273 (Moderna, USA), with 92% Sputnik V (Gamaleya, Russia), 63.09% AZD1222 (Oxford/AstraZeneca), 79% BBIBP-CorV (Sinopharm, China), 77.8% Covaxin (Barat Biotech, India), 66.9% Ad26. CoV.S “Janssen” (Johnson & Johnson, USA) and with 50.4% CoronaVac (Sinovac, China) [30, 31].

More than 1698 million doses have been administered in the Americas, completing with a complete immunisation schedule for more than 672 million people. The countries with the highest percentage of complete schemes per-100 inhabitants are the Cayman Islands (94.65%), Puerto Rico (92.09%), Chile (89.93%), Cuba (87.34%) Saba Island (81.27%). The most widely used vaccines in the continent were from Pfizer/BioNTech, Moderna, and Sinovac laboratories [32].

The impact of COVID-19 on the education, health, and well-being of the pediatric population has been significant. Because of this, immunisation coverage against COVID-19 in this population is necessary [33]. Although vaccination in children and adolescents is essential to reduce infection and transmission of the virus from the vaccinated to the susceptible person, in many countries, it is necessary to restore the stability of the educational system, mental and emotional health, and for their parents, due to the severe labour, economic and social problems caused by the closure of schools [33].

Despite the significant advances achieved with the various types of vaccines, only a few vaccines against COVID-19 have completed clinical trials in children (Table 1) and there are a further 28 underway [34]. Thus, the Pfizer/BioNTech vaccine is the first COVID-19 vaccine to be licensed for emergency use in children aged 5 to 17 years in the United States [35].

**Table 1:** Summary of clinical trials of COVID-19 vaccines in children

Preliminary efficacy (%)	Phase	Vaccine	Laboratory	Type	Age group (years)	References
100.0	3	BNT162b2	Pfizer/BioNTech	mRNA	12–15	[36]
90.7	2–3	BNT162b2	Pfizer/BioNTech	mRNA	5–11	[37]
100.0	1–2	BBIBP-CorV	Sinopharm	Inactivated virus	3–17	[38]
98.0	2	CTII-nCoV	Cansino	Non-replicant Viral Vector	6–17	[39]
96.0	1–2	CoronaVac	Sinovac	Inactivated virus	3–17	[40]
98.8	2–3	mRNA-1273	Moderna	mRNA	12–17	[41]

Some vaccines not yet approved by regulatory agencies such as the U.S. Federal Drugs Administration (FDA) or the WHO are being applied in some Latin American countries; for example, Chile approved the Sinovac COVID-19 vaccine for children over 6 years. El Salvador licensed COVID-19 vaccination for children aged 6 to 11 years. Argentina is vaccinating children as young as three years old with the Sinopharm COVID-19 vaccine. Ecuador's vaccination includes children as young as six years old with the Sinovac vaccine. Colombia offers COVID-19 vaccines from AstraZeneca, Moderna, Sinopharm, and Johnson & Johnson for children 12 years and older. Finally, Costa Rica is vaccinating from 12 years of age [42].

**Fig. 1:**
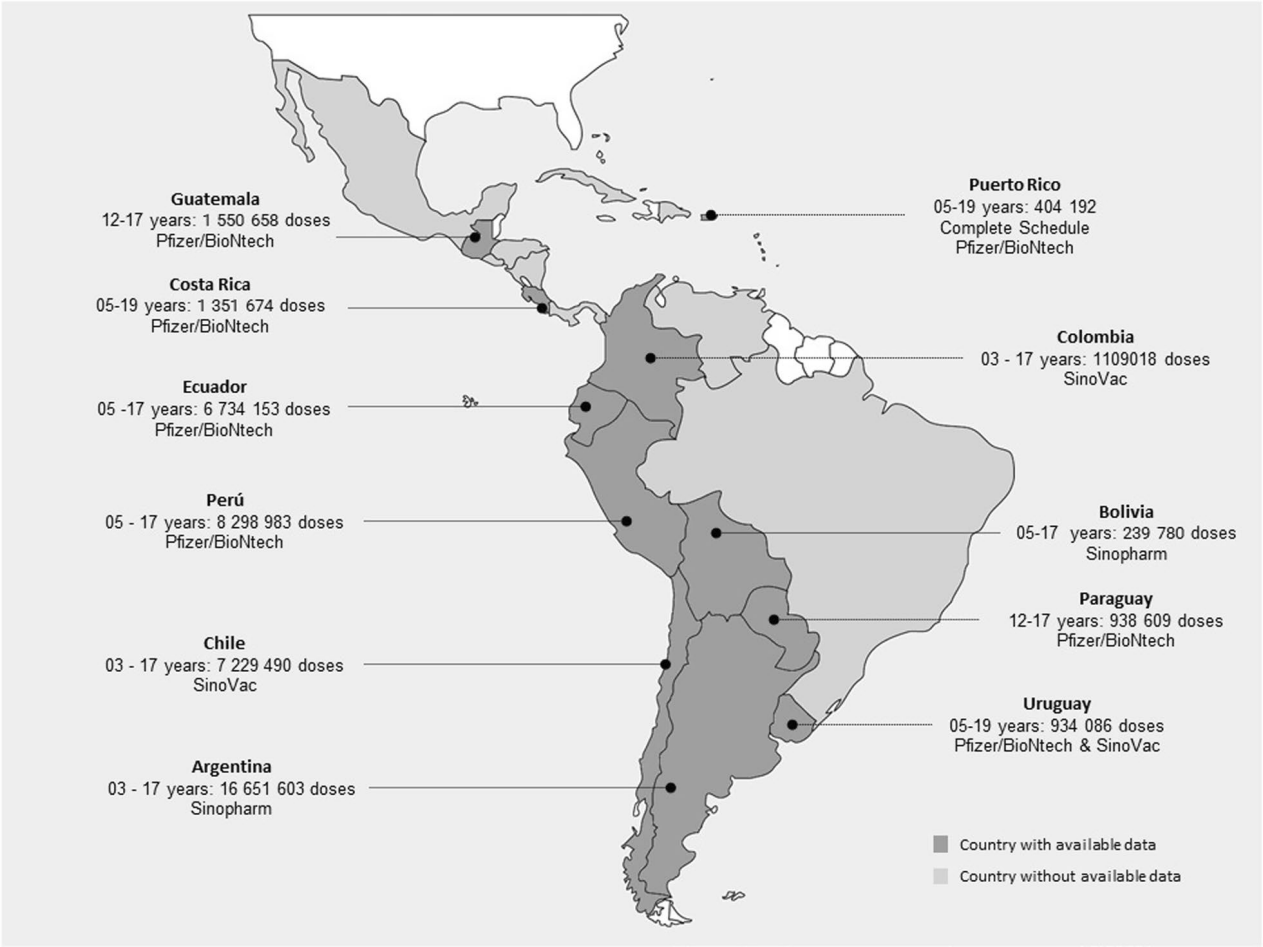
COVID-19 vaccines doses administered in children and adolescents in Latin America. Only countries with public data on vaccination in children and adolescents are included. Updated March 8, 2021

The distribution of vaccines against COVID-19 in Latin America is unevenly distributed. For example, Argentina has more than 16.6 million doses administered, Chile more than 7.2 million, and Ecuador more than 6.7 million. Most countries have started vaccination at 5 years of age. The most widely used vaccine in the region is the Pfizer/BioNTech vaccine (Fig. 1).

COVID-19 vaccines have proven effective and safe, demonstrating their effectiveness in reducing symptomatic disease, hospitalisations, and deaths; however, there are significant challenges, including approval by regulatory systems, and vaccine availability in all countries. In addition, there is still a lack of data on the efficacy of COVID-19 vaccines administered as a third (booster) dose, with some studies reporting that the booster dose increases the antibody and neutralising response, providing additional protection against SARS-CoV-2 infection for vaccines [43, 44].

It is very likely that, as the vaccination program progresses in the countries of Latin America, the target population will be modified to include the pediatric population which has been ignored in most countries, causing an increase in the incidence of infection in this population. It can be hoped that increased vaccination in the paediatric population will reverse this trend.
